# Fluorescent protein tagging as a tool to define the subcellular distribution of proteins in plants

**DOI:** 10.3389/fpls.2013.00214

**Published:** 2013-06-24

**Authors:** Sandra K. Tanz, Ian Castleden, Ian D. Small, A. Harvey Millar

**Affiliations:** ^1^ARC Centre of Excellence in Plant Energy Biology, The University of Western AustraliaPerth, WA, Australia; ^2^Centre of Excellence in Computational Systems Biology, The University of Western AustraliaPerth, WA, Australia; ^3^Centre for Comparative Analysis on Biomolecular Networks (CABiN), The University of Western AustraliaPerth, WA, Australia

**Keywords:** FP tagging, subcellular localization, database, Arabidopsis, subcellular proteomics

## Abstract

Fluorescent protein (FP) tagging approaches are widely used to determine the subcellular location of plant proteins. Here we give a brief overview of FP approaches, highlight potential technical problems, and discuss what to consider when designing FP/protein fusion constructs and performing transformation assays. We analyze published FP tagging data sets along with data from proteomics studies collated in SUBA3, a subcellular location database for Arabidopsis proteins, and assess the reliability of these data sets by comparing them. We also outline the limitations of the FP tagging approach for defining protein location and investigate multiple localization claims by FP tagging. We conclude that the collation of localization datasets in databases like SUBA3 is helpful for revealing discrepancies in location attributions by different techniques and/or by different research groups.

## Introduction

Plant systems are comprised of a complex network where organs, tissues, and cell types interact with each other. Each cell, in turn, is characterized by a comparably complex network of subcellular compartments that are morphologically and functionally different. Proteins located in these subcellular compartments often share similar attributes and play roles in defining the function of these distinct cellular environments. To understand how plant cells are functionally structured, we need to know where enzymes and regulatory proteins are located within the cell at certain points in development and under particular environmental conditions (Millar et al., 2009).

Different methods can be employed to help to determine a protein's intracellular location. Computational programs that can predict the subcellular location from the protein's nucleic acid sequence are useful but not conclusive (Richly and Leister, 2004; Heazlewood et al., 2005; Reumann, 2011). In addition, some proteins exist in multiple locations (Small et al., 1998; Carrie and Small, 2012) but only a few prediction programs deal with multiple locations effectively, such as ATP (Mitschke et al., 2009), Plant-mPLoc (Chou and Shen, 2010), WOLF PSORT (Horton et al., 2007), and YLoc (Briesemeister et al., 2010; for an overview of protein localization predictors see also Tanz and Small, 2011). *In vitro* uptake studies of an exogenously added protein into an isolated organelle has been a powerful tool for detailed studies of the import process but does not reproduce the complex intracellular environment and might not always reveal targeting preference between organelles (Rudhe et al., 2002; Chew et al., 2003). Immunolabeling of proteins in tissue sections, where specific antibodies recognize the native conformation of the protein, can be laborious and time-consuming and may not always be successful. This approach is also problematic when dealing with proteins with closely related sequences. Proteomic studies employing cell fractionation and mass spectrometry (MS) to identify peptides in the purified subcellular compartments result in large, information-rich datasets (Jaquinod et al., 2007; Reumann et al., 2007, 2009; Eubel et al., 2008; Mitra et al., 2009; Ferro et al., 2010; Olinares et al., 2010; Ito et al., 2011; Klodmann et al., 2011; Lee et al., 2011; Taylor et al., 2011; Zhang and Peck, 2011; Lundquist et al., 2012). However, MS can be technically challenging as contamination of the subcellular preparation with proteins from other parts of the cell is a frequent problem and low abundance, small and hydrophobic proteins can be missed employing this approach. Fusion of fluorescent protein (FP) coding sequences to the coding regions of genes of unknown location is relatively simple and fast and can be directed to specific proteins of interest, and as a result FP tagging has become the method of choice for many plant biologists.

FP tagging and subcellular proteomic studies have become the dominant tools for determining the location of a protein within the plant cell and provide complementary and independent information. However, these high-throughput approaches are prone to both false-negative and false-positive claims of protein location. In addition, the FP tagging approach defines a protein's targeting ability and defines a final location by accumulated fluorescent signal, while the subcellular proteomics approach determines, in steady-state, where the native protein accumulates in the cell. While it is expected that these two approaches should reveal matching results in most cases, they will not always agree even when the data from both methods is sound (Millar et al., 2009). Collating location data sets of different approaches in databases like SUBA (Heazlewood et al., 2007; Tanz et al., 2013) allows users to assess these data collectively and can expose discrepancies and conflicts in location attributions by different methods and/or by different research groups. In this report we review the current location data sets in SUBA3 (Tanz et al., 2013). Specifically, we focus on the subcellular location data by FP tagging and examine the broader reliability of these data compared to other experimental claims, discuss the limitations of the approach, and analyze localization claims by FP for the same protein in multiple locations.

## The FP tagging approach

### FP tagging in plants

Expression of the green fluorescent protein (GFP) from the jellyfish *Aequorea victoria* and its spectral variants within cells (Chalfie et al., 1994; Zacharias and Tsien, 2006) has stimulated many experiments to gain new insights into the organization of cellular metabolism and to better understand compartmentation of cells. FP tagging can now provide answers to the following questions: Where do proteins localize within the cell? Where do dynamic proteins move within the cell? How do individual proteins behave in response to developmental and environmental changes? However, heterologous expression of GFP in plant cells has not always been straightforward. Initially, GFP tagging was only successful in animal and fungal cells, whereas only poor GFP expression levels were observed in plant cells. This was due to the presence of a cryptic intron in the original jellyfish GFP sequence, which was incorrectly removed in plant systems. Modifications to the GFP codon sequence abolished the erroneous removal of part of the sequence and restored the expression of GFP in plant systems (Haseloff et al., 1997; Rouwendal et al., 1997).

Today, GFP and its derivatives and homologs (here collectively referred to as fluorescent proteins or FPs) are the most important fluorophores for plant cell biology and their use has been reported extensively in the literature (reviews include Hanson and Kohler, 2001; Ehrhardt, 2003; Dixit et al., 2006; Fricker et al., 2006; Berg and Beachy, 2008). Untargeted or “free” FPs are localized to the cytoplasm in plant cells but also go into the nucleus due to their small size. In addition, FPs have been targeted to all plant organelles using FP fusions incorporating location-specific signal sequences (Tian et al., 2004). In fact, a set of fluorescent organelle markers has been generated based on well-established targeting sequences (Nelson et al., 2007). All markers were generated with four different FPs in two different binary plasmids to allow for flexible combinations during co-localization studies (Nelson et al., 2007). The use of FPs to localize individual proteins is based on the ability to engineer FP fusions, with FP tagged onto the protein of interest, allowing it to be observed within intact tissue. FPs have even been used to tag viral proteins to investigate the interaction of such proteins with plant organelles (Lazarowitz and Beachy, 1999; Ueki and Citovsky, 2011). FP imaging does not require staining and allows analysis of cells in a relatively undisturbed, living state. This non-invasive way of monitoring localization and dynamics of proteins as well as there being no need for exogenous substrates or co-factors (Chalfie et al., 1994) are the main advantages of FP tagging.

A disadvantage with FP imaging, particularly in plants, has been the autofluorescence of cellular components such as cell walls and plastids, which may overlap with FP spectral signals (Deblasio et al., 2010). For example, interference by autofluorescence from the cell wall could be a problem for the localization of low abundant plasma membrane proteins. However, most modern confocal microscopes are now able to account for background autofluorescence and subtract it from FP signals based on the unique spectral profile of non-FP expressing reference images.

As increasing numbers of plant genomes are fully sequenced, high-throughput FP screens are being employed to identify gene function and regulatory networks (Cutler et al., 2000; Escobar et al., 2003; Tian et al., 2004; Koroleva et al., 2005; Marion et al., 2008). For example, a library of Arabidopsis cDNAs was generated and fused to the 3′ end of GFP. The library was then transformed into Arabidopsis *en masse* and the progeny screened for transgenic plants showing different subcellular localization patterns (Cutler et al., 2000). In a complementary study, open reading frame cDNA clones were GFP-tagged at their 3′ end and transformed cell cultures were screened for localization patterns (Koroleva et al., 2005). The Arabidopsis localizome project uses a recombineering-based gene tagging approach to generate FP fusion proteins in their chromosomal context (Zhou et al., 2011). A bacterial homologous recombination system is used to insert FP tags into genes of interest that are harbored by transformation-competent bacterial artificial chromosomes (TAC; Zhou et al., 2011). This ensures that all *cis*-regulatory sequences of a gene are included and because the genes are not amplified by PCR there is no limit to the size of a gene that can be tagged. Thus, this is a promising approach for the future that will eliminate many of the current problems encountered during FP tagging studies (see section Considerations with FP/Protein Fusions).

### Considerations with FP/protein fusions

The fusion of FP to enzymes often does not inhibit their catalytic activity and FP tagging is generally thought to be a “safe method” to determine the subcellular location of a protein. Indeed expressions of FP fusions of proteins have been reported to functionally complement knockout mutants (Sedbrook et al., 2002; Benkova et al., 2003; Kim et al., 2003). However, it is possible that in some cases the FP/protein fusion and the wild-type protein will differ in their subcellular locations leading to false positive results. Careful consideration is required where a protein is tagged, as the presence of the FP could hinder proper localization encoded by a transit sequence on the attached protein.

FP coding sequences are typically fused to either the 5′ or 3′ end of the coding region of a DNA sequence in question, generating N- or C-terminal FP fusions (Cutler et al., 2000; Huh et al., 2003). Alternatively, proteins can be tagged at a selected internal site, which has the advantage that targeting signals present at the 5′ or 3′ end of the coding region are not masked by the FP. For example, N-terminal fusions (FP is fused to the N terminus of the protein of interest) interfere with plastid and mitochondrial localization signals and are also likely to abrogate endoplasmic reticulum (ER) signal peptides. C-terminal fusions (FP is fused to the C terminus of the protein of interest) may also cause many proteins to mislocalize, particularly peroxisomal proteins. In addition, C-terminal fusions could mask stem-loop structures in the 3′ part of the coding sequence and the 3′ untranslated region, which are necessary for the accurate localization of certain mRNAs (Chartrand et al., 1999). N- or C-terminal fusions may also interfere with posttranslational modification sites, such as myristylation or farnesylation sites important for membrane targeting. Indeed, some plasma membrane proteins failed to localize to the plasma membrane using N- or C-terminal tags but internally tagged proteins localized correctly (Sedbrook et al., 2002; Gardiner et al., 2003; Tian et al., 2004). In addition, more and more multi-targeted proteins are being identified. For example, proteins with peroxisomal targeting signals and chloroplast or mitochondrial transit peptides have only been identified when analyzed with separate N- and C-terminal fusion constructs (Carrie et al., 2008; Hooks et al., 2012). Thus, for correct localization it is crucial to examine N- and C-terminal FP fusion constructs and/or internally tagged proteins.

Similarly, the length of a protein sequence for fusion with an FP needs to be considered. Using the full-length sequence of a protein is desirable; however, some genes might be too long to be easily cloned into an expression vector and thus partial sequences are frequently used for localization by FP tagging. Most plastid or mitochondrial targeting sequences are located at the N-terminus and the N-terminal ~100 amino acids are generally sufficient for correct subcellular localization. However, in this case a possible second C-terminal or internally located targeting sequence might be missed, as in the case of multi-targeted proteins (Carrie et al., 2009; Hooks et al., 2012).

The promoter used in front of an FP fusion construct also needs to be considered. Often the CMV 35S promoter is used instead of the native gene promoter, which could lead to higher expression levels of the fusion construct than for the endogenous protein, and subsequently could lead to mistargeting. This could particularly affect nuclear-encoded proteins targeted to organelles, where high protein abundance could result in incomplete import. Theoretically this might also account for some false claims of dual targeting of proteins between the cytoplasm and various organelles.

In addition, the fused FP could be the reason for a conformational modification in the attached protein and a localization signal could become active, which is normally isolated in the absence of FP or when it is lacking some endogenous ligand. Also, the abundance of the fused FP may be very different from the native protein, leading to mislocation, aggregation, metabolic disturbance or the like.

### Considerations with transformation assays during FP tagging

FP fusion constructs can be introduced into plant cells for transient assays or stably expressed in transgenic plants. With the latter, many different cell types can be investigated in which the FP/protein fusion is expressed, while not all cell types are suitable for transient expression. In addition, cell damage often occurs during DNA uptake in transient assays and inconsistent amounts of FP fusion constructs can be delivered into the cells. Thus, it is more reliable overall to analyse healthy stable transformants to define protein location by FP. However, the simplicity and speed of transient assays makes them a very valuable tool, especially when considering the extra labor and analysis it takes to generate and test stable transgenic plants. Onion epidermis is a favorite material for biolistic transient assays, because of its clear cytoplasm and single layer of living cells. Similarly, Arabidopsis cell culture, Arabidopsis seedlings and young detached leaves have also been successfully used in transient assays. Following particle bombardment with various constructs, cellular compartments such as ER, Golgi, vacuole, mitochondria, plastids and plasma membrane can all be labeled by different transiently expressed FP fusions in Arabidopsis (Nelson et al., 2007). Other popular transient expression methods include the protein expression in isolated protoplasts by electroporation or using polyethylene glycol (Miao and Jiang, 2007; Yoo et al., 2007) and the Agrobacterium-mediated infiltration in *Nicotiana benthamiana* (Yang et al., 2000) or Arabidopsis leaves (Tsuda et al., 2012).

## Analysis of FP tagging data in SUBA3

### The reliability of FP localization data

Given that various approaches have been used to define the location of proteins, and each has its own drawbacks, it is important to ask: What is the reliability of the FP tagging approach? In an attempt to answer this question we have analyzed subcellular localization data in SUBA (Heazlewood et al., 2007; Tanz et al., 2013). At the time of writing, SUBA3 contains a total of 3788 entries based on FP tagging studies from 1074 different publications, representing 2477 unique proteins. Of these, 443 proteins have been localized at least twice independently by FP, and for 375 proteins the independent FP localizations agree. Thus, for 85% of cases, the FP data are internally consistent, whereas they disagree in the cases of 123 proteins (28%). For 13% of proteins, the FP localization of one publication has been shown to agree with a second publication, and shown to disagree with a third publication; these proteins count toward both groups. Additional data based on subcellular MS-based proteomics from 122 different publications add 22,191 entries on 7685 distinct proteins. Calculating the percentage of FP tagging and MS agreements/disagreements for proteins for which both FP tagging and proteomics data are available shows that 61% of the data agree and 39% disagree. The remaining 1593 FP entries are not confirmed nor do they disagree with MS data because no independent subcellular proteomics data relating to these proteins have been published to our knowledge. Analyzing the FP data set further and comparing it to data from subcellular MS-based proteomics reveals that 849 out of 2996 FP protein claims agree with proteomics data (Table 1). The number of protein claims (2996) is different to the number of unique proteins (2477) because it includes cases where the same protein has been found in multiple compartments and thus accounts for multiple entries, and it is also different to the total FP entries (3788) as a protein is only counted once per location regardless how many researchers have found it in the same location. In these 849 cases, the protein's targeting ability tested by FP tagging agrees with the protein's accumulation tested by subcellular MS and we can be confident of the location claim and how the protein got there. On the contrary, for 554 FP claims a different location has been reported by MS studies. Thus, published disagreement of subcellular location exist for these FP claims and the protein's targeting ability appears to disagree with the claimed location of the protein's accumulation.

**Table 1:** **Number of localizations by FP tagging for each of the 11 subcellular compartments in SUBA3**.

**Intracellular location**	**FP localizations**	**FP localizations confirmed by MS**	**FP localizations contradicted by MS**	**FP and MS localizations agree/disagree (% of proteins with both FP and MS data)**	**FP localizations neither confirmed nor contradicted**
Cytoskeleton	63	0	24	0/100	39
Cytosol	514	119	101	54/46	294
ER	189	31	56	36/64	102
Extracellular	40	9	10	47/53	21
Golgi	145	42	57	42/58	46
Mitochondrion	312	90	54	63/37	168
Nucleus	779	95	149	39/61	535
Peroxisome	130	59	33	64/36	38
PM	245	122	17	88/12	106
Plastid	486	248	34	88/12	203
Vacuole	93	34	18	65/34	41
Total	2996	849	554	61/39	1593

Also shown are the numbers of FP localizations that overlap with MS localization and are thus confirmed by this approach, the numbers of FP localizations that disagree with MS localizations, the percentage of agreements and disagreements of FP with MS localizations for proteins for which FP tagging and proteomics data are both available, and the numbers of FP localizations that are neither confirmed nor contradicted by MS data. Data sets were extracted from the SUBA3 database (http://suba.plantenergy.uwa.edu.au).

Abbreviations: FP, fluorescent protein; MS, mass spectrometry; ER, endoplasmic reticulum; PM, plasma membrane.

A detailed list of the existing FP data for each of the 11 compartments in SUBA3 is shown in Table 1, along with the independent confirmations and disagreements by published subcellular proteomics data. For most of the compartments, the agreements between the claims for localization by FP tagging and subcellular MS lie between 36% and 65% for proteins with both FP and MS data available (Table 1). However, for two compartments, namely plastid and plasma membrane, 88% of proteins for which FP and MS data are available show an agreement and only 12% of FP data do not agree with the MS localization data (Table 1). The relatively high discrepancy between FP and MS data for most of the other compartments (35–64%, Table 1), likely highlights technical problems in false positive rates with both the MS and FP tagging approaches but further analysis will be required to confirm this.

The three organelles plastid, mitochondrion and peroxisome were chosen as examples to closer investigate the proteins for which a disagreement between FP and MS data has been observed.

#### Plastid

A total of 486 proteins have been localized to the plastid by FP tagging (Table 1). From these, the published plastid FP localizations of 34 proteins appear to disagree with the locations claimed by proteomics studies (Supplementary Table 1). For eight of these proteins, additional FP location data for the same proteins agree with MS location claims and thus the whole FP data set does not strictly disagree with the proteomics (Supplementary Table 1, AGIs with asterisk). Investigating the 34 proteins more closely reveals that seven proteins are known to be dual-targeted or dynamic so here the two data sets may both be correct (Supplementary Table 1, yellow). Another eight proteins clearly have a function in the plastid with two of these located in a second compartment other than the one determined by MS (Gao et al., 2003; Lurin et al., 2004; Murcha et al., 2007; Yu et al., 2008; Sun et al., 2010; Skalitzky et al., 2011). Thus, the disagreements are due to technical issues with the MS approach and could result from contamination of these proteins in sample preparations of other subcellular structures (Supplementary Table 1, blue). One of these proteins is OEP16 (At4g16160), localized by FP tagging to the plastid and by MS to the cytosol, but it has been confirmed by *in vitro* imports to be targeted to plastids and not to mitochondria, unlike the mitochondrial isoforms of this protein family (Murcha et al., 2007). The disagreement is likely due to be an error or contamination in the MS approach (Supplementary Table 1, blue). One protein (Complex I subunit At2g02510) clearly functions in the mitochondrion (Brugiere et al., 2004; Meyer et al., 2008; Klodmann et al., 2011), and the disagreement in localization is due to technical issues with the FP tagging approach (Supplementary Table 1, green). These include artifacts that may result from the foreign passenger protein affecting the targeting ability of the protein of interest, such as difference in abundance of the fusion protein, conformational changes or activation of a localization signal in the attached protein (see section Considerations with FP/Protein Fusions). The remaining 18 proteins are either unknown multi-targeted proteins located to the plastid and other compartments in the cell or the disagreement between FP and MS data is due to limitations of one or both approaches.

An interesting example for when experimental data appear to disagree but when in fact they actually complement each other is alanyl-tRNA synthetase (At1g50200). FP tagging studies found this protein to be targeted to plastids and mitochondria, whereas proteomics studies found it in the cytosol (Supplementary Table 1). Analysis of the transcription of the gene showed the presence of two translation initiation codons (Mireau et al., 1996). Translation from the upstream AUG generates an N-terminal extension with features that target the protein to the mitochondrion and plastid, whereas most ribosomes initiate on the downstream AUG to give the shorter polypeptide corresponding in size to the cytosolic enzyme (Mireau et al., 1996). Examining the peptides identified in the cytosolic MS study (Ito et al., 2011) showed that all the cytosolic peptides significantly matching to At1g50200 (see Ito et al., 2011; Supplementary Table 1, protein hit number 68) are downstream of the second start methionine. Thus, alanyl-tRNA synthetase is only expressed at low levels in mitochondria and plastids, which explains why MS studies have not found it in these organelles but only in the cytosol and why FP studies, using the full-length sequence, have only found it in plastids and mitochondria but not in the cytosol.

#### Mitochondrion

Examining the 54 proteins that have been localized to the mitochondrion by FP tagging but elsewhere by subcellular MS studies shows that as many as 37 of these have additional FP data that agree with MS locations (Supplementary Table 1, AGIs with asterisk). Twenty six of these 54 proteins are known dual-targeted or dynamic proteins (Supplementary Table 1, yellow). In both cases no strict disagreement exists. Eight proteins are clearly localized to and have a function in the mitochondrion as defined by FP tagging (six of these are additionally targeted to a second compartment different to the one defined by MS) and the location disagreements are due to technical issues with the MS approach (Supplementary Table 1, blue) (Souciet et al., 1999; Escobar et al., 2003; Michalecka et al., 2003; Duchene et al., 2005; Murcha et al., 2007; Carrie et al., 2008, 2009; Palmieri et al., 2009). Another seven proteins are clearly not located in the mitochondrion but function in the plastid (Hjelmstad and Bell, 1990; Froehlich et al., 2003; Asano et al., 2004; Chew et al., 2004; Friso et al., 2004; Kleffmann et al., 2004; Peltier et al., 2004; Giacomelli et al., 2006; Peltier et al., 2006; Rutschow et al., 2008; Zybailov et al., 2008; Ferro et al., 2010; Olinares et al., 2010; Granlund et al., 2011), and here the disagreement in location is due to technical issues with the FP tagging approach (Supplementary Table 1, green). The remaining 13 proteins are either unknown multi-targeted proteins or the disagreement is due to limitations of the FP tagging or the subcellular MS approach.

#### Peroxisome

One hundred and thirty proteins are localized to the peroxisome by FP tagging, of which 33 are localized elsewhere by proteomic studies (Table 1). Eight of these have additional FP data that agree with MS locations (Supplementary Table 1, AGIs with asterisk). Eight of the 33 proteins are known to be dual-targeted or dynamic proteins and the two data sets do not necessarily disagree (Supplementary Table 1, yellow). Three proteins are clearly localized to the peroxisome and have a function in the peroxisome (Cutler et al., 2000; Carrie et al., 2008, 2009) as defined by FP tagging [with two of them, a substrate carrier (At3g55640) and a NAD(P)H dehydrogenase (At4g28220), also localized to another compartment different to the one determined by MS], and the location disagreement is due to technical issues with the MS approach (Supplementary Table 1, blue). Four proteins are either unknown multi-targeted proteins or the location difference is due to limitations of one or both approaches (Supplementary Table 1, no color). However, about half of the location discrepancies between the two methods are due to technical issues with the FP tagging approach as most proteins are most likely not localized to the peroxisome and have functions elsewhere in the cell (Supplementary Table 1, green).

### Multiple localization claims by FP tagging

The redundancy that is apparent between 2996 FP localizations in Table 1, but 2477 unique proteins localized by FP tagging, is either due to multiple locations claimed by single literature reports or independent reports claim different locations for a single protein. Examples for the former include dual-targeted proteins to chloroplasts and mitochondria (Peeters and Small, 2001; Carrie and Small, 2012), to mitochondria and peroxisomes (Carrie et al., 2009), and to mitochondria and nucleus (Carrie et al., 2009; Hammani et al., 2011).

Analyzing only the FP tagging data in SUBA3 generated a total of 739 claims where proteins are localized to two different locations (Table 2). The 739 claims comprise 545 distinct proteins that have been localized to at least two different cellular compartments by FP tagging. A paired matrix of these data displays these dual localization claims for each possible subcellular compartment combination (Table 2). There is typically 1–20% overlap between any two subcellular proteomes. However, a 31% and 46% overlap exists between nucleus and cytosol and a 20% and 32% overlap between plastid and mitochondrion (Table 2). This can be partially explained by dynamic proteins that can move between nucleus and cytosol and proteins that are dual-targeted to these compartments. No doubt, the FP tagging approach has its limitations and some false positive results must also be contributing to these overlaps. Furthermore, a dual localization to the nucleus and cytosol can be due to FP artifacts, including GFP localizing by itself to the cytosol and the nucleus, which can generate false positive results to these two compartments.

**Table 2:** **A paired matrix showing dual FP localization claims for each possible subcellular compartment combination**.

**FP localization**	**Cytoskeleton**	**Cytosol**	**ER**	**Extracellular**	**Golgi**	**Mitochondrion**	**Nucleus**	**Peroxisome**	**PM**	**Plastid**	**Vacuole**
**Cytoskeleton**	63										
**Cytosol**	11	514									
**ER**	3	15	189								
**Extracellular**	1	3	1	40							
**Golgi**	3	21	28	2	145						
**Mitochondrion**	2	12	4	0	2	312					
**Nucleus**	12	238	13	4	1	8	779				
**Peroxisome**	2	11	2	0	1	10	5	130			
**PM**	8	45	21	14	22	3	23	0	145		
**Plastid**	2	16	10	0	2	100	7	8	3	486	
**Vacuole**	1	5	10	1	10	0	2	2	7	2	93

In total, 739 claims are listed, comprising 545 distinct proteins that have been localized to at least two different cellular compartments by FP tagging. The matrix diagonal shows the set of proteins claimed in each compartment. In the matrix below the diagonal, the two-way comparisons of claims for proteins to be present in different compartments are shown. Data sets were extracted from the SUBA3 database (http://suba.plantenergy.uwa.edu.au).

Abbreviations: FP, fluorescent protein; ER, endoplasmic reticulum; PM, plasma membrane.

Of the 739 claims where proteins are localized to two different locations, 80% (595 dual claims) are by the same literature reports. These comprise 491 proteins and because the dual location is reported by the same publication these are presumably dual- or multi-targeted proteins. 20% of these claims (representing 105 proteins) demonstrate a conflict in the literature (as they appear as different publications that contradict each other) and may highlight problems associated with the use of different FP tagging approaches. However, this set could also include biological discoveries such as identification of an unknown dual-targeted protein or showing dynamic proteins that move around in the cell in different cell types or treatments.

As examples for further investigation, the dual FP localization claims for mitochondrion/plastid, mitochondrion/peroxisome, and plastid/peroxisome were chosen.

#### Mitochondrion and plastid

Examining the literature references of the 100 proteins that have been located by FP tagging to the plastid and mitochondrion (Table 2) reveals that the dual localizations of 92 proteins are described in the same literature reports and these proteins are presumably dual-targeted (Supplementary Table 2, “Y”). Indeed when investigating the function of these proteins, many are known dual-targeted proteins (Supplementary Table 2, yellow). Nevertheless, four proteins are likely to be only located to the mitochondrion (Supplementary Table 2, orange) and another eight only located in plastids (Supplementary Table 2, green). Thus, here the apparent dual location is due to technical issues with the FP tagging approach that could involve a difference in abundance of the fusion protein or conformational changes leading to activation of a localization signal in the attached protein (see section Considerations with FP/Protein Fusions). For eight proteins a literature conflict exists and independent reports claim mitochondrial and plastid locations for a single protein. These proteins are either dual-located proteins, or the dual localizations are false positives due to technical problems with the FP tagging approach. In fact, based on their function and from independent literature reports, two of these eight proteins are already known dual-located proteins [dynamin 3A (At4g33650) and lon1 protease (At5g26860); Supplementary Table 2, yellow] and four are known to be located in the plastid only (Supplementary Table 2, green) indicating an issue with the FP approach.

#### Mitochondrion and peroxisome

Ten proteins have been localized to mitochondria and peroxisomes by FP tagging (Table 2) and the dual-locations of all ten proteins are each reported by the same publication, indicating all ten proteins are probably truly dual-targeted (Supplementary Table 2, “Y”). In fact, more than half of the proteins are known dual-targeted proteins from other literature (Supplementary Table 2, yellow).

#### Peroxisome and plastid

Of the eight distinct proteins that have been localized to the peroxisome and plastid by FP tagging, five proteins are presumably dual-targeted (same publication; Supplementary Table 2, “Y”), of which two are known dual-targeted proteins based on the function (Supplementary Table 2, yellow). The remaining three proteins demonstrate a conflict in the literature (Supplementary Table 2, “N”), of which two are clearly only located in the plastid [Rubisco small chain 1A (At1g67090; Parry et al., 2003) and chaperonin 20 (At5g20720; Carrie et al., 2009)] and the multiple localizations of these proteins likely represent technical problems with the FP tagging method (Supplementary Table 2, green). The third is the same dynamin 3A (At4g33650) noted above; the plastid claim for this protein by FP pre-dated the dual-targeting claim in mitochondria and peroxisomes by 6 years. While an explanation of why a plastid FP location was found has not been provided, the weight of genetic and other evidence appears to suggest this is a technical problem with the FP claim of the plastid location (Mano et al., 2004).

## Conclusions

FP tagging with its rapidity and simplicity has become a very important tool for plant biologists to localize proteins at a subcellular level. The analysis of the FP-tagging localization dataset along with the subcellular proteomics data, both available in SUBA3, has revealed subcellular compartments where up to 88% the FP localizations have been confirmed by subcellular proteomics for proteins for which both data are available. Thus, here the protein's targeting ability agrees with its observed protein's accumulation. The more data become available in the future, the better the coverage of each subcellular proteome and the higher the agreement between different methods is likely to be. However, with more data the number of disagreements between methods will also increase. Examining the number of existing disagreements between FP tagging and MS for the individual subcellular compartments has already exposed discrepancies in location attributions between the two methods as high as 39% of the total FP datasets for proteins for which both FP and MS data are available. Such a high discrepancy highlights problems with both the MS and FP tagging approaches, which are evident when looking closely at the organelle examples of the plastid, mitochondrion and peroxisome. Apart from the technical issues and limitations of both approaches, the disagreements can also be due to unknown biology (dual-targeted proteins or dynamic proteins). Similarly, investigating the localization disagreements within the FP tagging method showed that the majority of multiple localization claims (80%) are due to multi-targeted proteins. The remaining 20% demonstrate a conflict in location attributions by different research groups and are possibly due to problems with the FP tagging approach, but may in some cases include dynamic proteins or unknown dual-targeted proteins. To be able to assess such localization data and draw conclusions about the reliability of localization methods and expose their limitations, collation of published results in databases like SUBA3 is extremely helpful. The intersections where existing data disagree could be avenues for new biological discoveries to be made.

### Conflict of interest statement

The authors declare that the research was conducted in the absence of any commercial or financial relationships that could be construed as a potential conflict of interest.
